# Atypical granuloma formation in *Mycobacterium bovis*-infected calves

**DOI:** 10.1371/journal.pone.0218547

**Published:** 2019-07-15

**Authors:** Jacobo Carrisoza-Urbina, Elizabeth Morales-Salinas, Mario A. Bedolla-Alva, Rogelio Hernández-Pando, José A. Gutiérrez-Pabello

**Affiliations:** 1 Laboratorio de Investigación en Tuberculosis Bovina, Departamento de Microbiología e Inmunología, Facultad de Medicina Veterinaria y Zootecnia, Universidad Nacional Autónoma de México, Mexico City, Mexico; 2 Departamento de Patología, Facultad de Medicina Veterinaria y Zootecnia, Universidad Nacional Autónoma de México, Mexico City, Mexico; 3 Unidad de Patología Experimental, Departamento de Patología del Instituto Nacional de Ciencias Médicas y Nutrición Salvador Zubirán, Mexico City, Mexico; The University of Georgia, UNITED STATES

## Abstract

Bovine tuberculosis is a chronic inflammatory disease that causes granuloma formation. Characterization of granulomatous lesions of *Mycobacterium bovis* (*M*. *bovis)* experimentally infected cattle has helped to better understand the pathogenesis of this disease. However, few studies have described granulomas found in *M*. *bovis* naturally infected cattle. The aim of this work was to examine granulomas from Holstein-Friesian cattle naturally infected with *M*. *bovis* from a dairy basin located in the central region of Mexico. Tissue samples from thirty-two cattle with lesions suggestive of tuberculosis were collected post-mortem. Fifteen of the 32 sampled animals (46.8%) were 4 months of age or younger (calves), whereas the rest (53.2%, 17/32) were over one year old (adults). Macroscopic lesions suggestive of tuberculosis were found in the mediastinal lymph node chain of all animals (32/32). From the 1,143 granulomatous lesions that were microscopically analyzed, 34.6% (396/1143) were collected from adult animals and subsequently classified according to the nomenclature suggested by Wangoo *et al*., 2005. Surprisingly, lesions from calf tissues showed an atypical pattern which could not be fitted into the established developmental stages of this classification. Granulomatous lesions found in calves covered most of the affected organ, histologically showed large necrotic areas with central calcification, absence of a connective tissue capsule, and few giant cells. Also, there was a higher percentage of lesions with acid-fast bacilli (AFB) when compared to studied granulomas in adults. Growth of *Mycobacterium spp* was detected in 11 bacteriological tissue cultures. Genotypic identification of *M*. *bovis* was performed by DNA extraction from bacterial isolates, formalin-fixed and paraffin-embedded (FFPE) tissues and samples without bacterial isolation. *M*. *bovis* was detected by PCR in 84.3% (27/32) of the studied cases; whereas other AFB were observed in tissues of the remaining sampled animals (5/32). Our results describe atypical granuloma formation in calves 4 months of age or younger, naturally infected with *M*. *bovis*. These findings contribute to better understanding the physiopathology of *M*. *bovis* infection in cattle.

## Introduction

Bovine tuberculosis (bTB) is a disease caused by *M*. *bovis*. It affects a wide range of mammals including humans, creating public health problems as well as economic losses for the livestock industry, due to factors such as low milk production, organ condemnation during *post-mortem* examination, and costs of eradication programs. It has been estimated that there are over 50 million infected cattle worldwide, generating losses of close to 3 billion dollars per year [1–4]. bTB is characterized by a chronic inflammatory process, which mainly affects the lungs and lymph nodes associated with the respiratory system, characterized by formation of caseous and necrotizing granulomas. Pathological changes associated with the infection reflect an interaction between immune responses and pathogen virulence factors [5].

Granulomas are considered the pathognomonic lesions of tuberculosis. Their formation represents an attempt by the host to isolate and contain mycobacteria, as well as to limit further surrounding tissue damage by dampening chronic inflammation [6]. Granulomatous lesions caused by *M*. *bovis* have been extensively studied to determine mycobacterial growth control mechanisms by the host, as well as pathogen survival ability, in order to better understand pathogenesis of the disease [7]. Granulomas have been classified in *M*. *bovis* experimentally infected cattle according to morphological criteria such as degree of necrosis and mineralization, and presence of a connective tissue capsule [8]. Lesions and tissues surrounding granulomas have also been examined [9–11]. In addition, techniques such as immunohistochemistry (IHC) or laser capture microdissection in combination with real time quantitative polymerase chain reaction have been implemented to identify immunological cell composition of lesions [8,9]. However, little is known about macroscopic and microscopic characteristics of granulomas derived from *M*. *bovis* naturally infected cattle. Therefore, the aim of this study was to examine granulomatous lesions of *M*. *bovis* naturally infected Holstein-Friesian cattle. The four stages of granuloma formation previously described by Wangoo *et al*., (2005) [8] were identified in 17 animals over one year of age. However, all granulomas found in tissues from 15 calves 4 months of age or younger, showed atypical structures.

## Material and methods

### Ethics statement

All procedures were reviewed and approved by the Ethics and Animal Welfare Committee of the Facultad de Medicina Veterinaria y Zootecnia, Universidad Nacional Autónoma de México (CICUA, FMVZ-UNAM), and complied with the Mexican guidelines for animal research (JAGP-074).

### Sample collection

Tissue samples presenting lesions suggestive of tuberculosis were collected with owner consent at post-mortem examination of animals. All cattle died from conditions that did not include tuberculosis. Animals were located at the central region of Mexico. Prevalence of bovine tuberculosis in this dairy basin is reported as being higher than 16% [12].

### Macroscopic pathology

During necropsy examination the carcass was externally and internally inspected, and organ systems were removed. Samples of lymph nodes, lung tissue and individual organs presenting lesions suggestive of tuberculosis were collected. Tissues with lesions were divided in two halves; one was used for histopathological studies and the other for bacteriological cultures.

### Histopathological analyses

Tissue segments presenting lesions suggestive of tuberculosis were fixed in 10% formaldehyde and paraffin-embedded. Serial sections of approximately four microns were used for staining with Hematoxylin and Eosin (H&E), Masson's trichrome, Ziehl Neelsen (ZN) and Von Kossa. Stained sections were examined under a CxL Labomed photonic microscope. Granulomatous lesions identified within H&E stained sections were classified in four developmental stages [8]. Acid-fast bacilli were quantified in ZN stained sections, using the 0–3 range scale where 0 = devoid of bacilli; 1 = 1–10 bacilli; 2 = 11–50 bacilli; and 3 = >50 bacilli [13]. Masson and Von Kossa trichromic stained sections were used to determine presence of connective tissue and degree of calcification respectively.

### Bacteriological isolation

Bacteriological isolation of samples followed biosecurity conditions of Petroff's decontamination method [14]. Approximately 2 cm^2^ of tissue from samples were individually macerated in a mortar with previously sterilized sand. Sand was decontaminated with 10% hydrochloric acid and 2 M sodium hydroxide. Macerates were centrifuged at 3,000 g for 20 min, supernatants were discarded and sediment was inoculated on Lowenstein Jensen and Stonebrink culture medium. Culture tubes were subsequently placed in the incubation chamber at 37°C for a period of 8–12 weeks. Media in samples were weekly assessed for microbial contamination. If contamination was deemed positive, tubes were discarded and the procedure repeated.

### Extraction of genomic DNA of *Mycobacterium spp*

Genomic DNA was extracted from: (a) eleven cultures with suggestive growth of *Mycobacterium spp*., (b) nine tissues from negative bacteriological growth cases, and (c) twelve formalin-fixed paraffin-embedded (FFPE) tissues that had histopathological lesions compatible with tuberculosis and presence of AFB. Samples were placed in 400 μl of TE solution (100 mM tris-HCl, 10 mM EDTA, pH 8), and inactivated at 80°C for 30 min. Fifty μl of lysozyme (10 mg/ml) was then added (SIGMA-Aldrich USA) and samples were incubated at 37°C for 16 h. A total of 75 μl of 10% SDS and 50 μl of K-proteinase (1 mg/ml) (SIGMA-Aldrich, USA) was subsequently added and samples were further incubated at 65°C for 10 min. Finally, 100 μl of CTAB (N-cetyl-N, N, N-trimetyl ammonium bromide) (SIGMA-Aldrich, USA) and 100 μl of 5 M NaCl was added and again incubated at 65°for 10 additional min. DNA was isolated by use of 700 μl of chloroform/isoamyl alcohol in a 24:1 ratio (SIGMA-Aldrich, USA). The liquid phase was recovered by centrifuging at 12,000 g for 5 min, and this step was repeated with one ml of chloroform/isoamyl alcohol. This phase was subsequently precipitated with 0.7 ml of absolute isopropyl alcohol (SIGMA-Aldrich, USA) and washed with 1 ml of 70% ethyl alcohol. The DNA pellet was then resuspended in 50 μl of nuclease-free water (GIBCO, Auckland, NZ) [15].

For DNA extraction from FFPE tissues, 12 micron sections were obtained by use of a microtome, which was cleaned with 70% alcohol between slices to avoid cross-contamination of samples. One ml of xylol was added to each tissue section which was then vortexed and incubated for 5 min. Xylol was subsequently decanted and tissue section was washed twice with absolute ethyl alcohol, allowed to dry and resuspended in 400 μl of TE and of lysozyme was then added continuing with the procedure previously described [15].

DNA concentration and purity were assessed by spectrometry (D.O. at 260/280 nm) using a Nanodrop spectrophotometer (ND-1000). Integrity of samples was evaluated by electrophoresis with a 0.7% agarose gel for 60 min at 80 volts.

### Polymerase chain reaction (PCR)

A nested PCR was performed to amplify the mpb70/m22 genes, which identifies members of the *Mycobacterium tuberculosis* complex. In addition, an endpoint PCR of the RD9 and RD4 genes was used to differentiate *M*. *bovis*, following manufacturer specifications of a commercial kit (TopTaq Master Mix Kit). A total of 50 ng of DNA sample were added to each reaction. Amplification protocols were performed on a thermal cycler (Thermo-Hybaid, USA). For conventional PCR, the used primers for the mpb70 gene that amplify a product of 372 bp were: mpb70 F (5’-GAACAATCCGGAGTTGACAA-3’) and mpb70 R (5’-AGCACGCTGTCAATCATGTA-3’). In addition, for a second reaction to obtain a 208 bp product from the same gene, the M22 F (5’-GCTGACGGCTGCACTGTCGGGC-3’) and M22 R (5’-CGTTGGCCGGGCTGG TTTGGCC-3’) primers were used. For the RD9 gene, selected primers were RD9 F (GTGTAGGTCAGCCCCATCC), RD9 I (CAATGTTTGTTGCGCTGC) and RD9 R (GCTACCCTCGACCAAGTGTT), with a product of 333 bp for *M*. *tuberculosis* and 206 bp for *M*. *bovis*. and for RD4 gene the primers were RDF (ATGTGCGAGCTGAGCGATG), RD4 I (TGTACTATGCTGACCCATGCG) and RD4 R (AAAGGAGCACCATCGTCCAC), with a product of 268 bp for *M*. *bovis* and *M*. *bovis* BCG, for the rest of the members of the Mycobacterium tuberculosis complex a product of 172 bp is amplified [16–18]. Results of the PCR reactions were separated by electrophoresis in a 2% agarose gel, with TAE solution (40 mM Tris-acetate, 1 mM EDTA pH 8), SYBR Green (S9430 SIGMA-ALDRICH) for DNA staining, and a molecular weight marker of 100–1,500 bp (Ready to use DDL-001). The reaction product was visualized using a photo documenter (Gel Logic 200 Imaging System, Kodak, UK).

### Statistical analyses

For statistical analyses the PASW Statistics 18 and the GraphPad prism 7.0 programs were used. Chi square test and Spearman correlation coefficient were used to identify if bacilli quantification scale was dependent on lesion stage, and if there was a correlation between variables.

## Results

### Identification of extensive granulomatous lesions in calves when compared to adult cattle

Tissue samples were collected post-mortem from 32 cattle that presented with macroscopic lesions suggestive of tuberculosis, later confirmed by histopathology. Mediastinal lymph nodes of all studied cattle presented suggestive tuberculous lesions macroscopically. Also, granulomas could be seen in lungs of 50% (16/32) of the animals. Lesions exclusively located in mediastinal lymph nodes were observed in 15.6% (5/32) of cattle, whereas the remaining animals presented lesions in more than one organ. It is noteworthy that 53.2% (17/32) of the sampled animals were over one year old, while the other 46.8% (15/32), were calves with ages ranging from one week to four months **(Table 1)**. Since differences in macroscopic and microscopic characteristics of lesions between these two age groups were observed, our results were accordingly divided in: a) cattle over one year old (adults) and b) one week to four-month-old animals (calves).

**Table 1:** Distribution of granulomatous lesions in cattle naturally infected with *M*. *bovi*.

Case number	Age	Sex	Organs with lesions suggestive of tuberculosis						
			Lungs	Retropharyngeal LN	Mediastinal LN	Hepatic LN	Liver	Retromammary LN	Mesenteric LN
1	4 Years	F		**+**	**+**			**+**	
2	4 Years	F			**+**				
3	2 Years	F			**+**				
4	3 Years	F	**+**		**+**				
5	3 Years	F	**+**		**+**				
6	5 Years	F			**+**			**+**	
7	5 Years	F			**+**				
8	3 Years	F	**+**		**+**	**+**			**+**
9	6 Years	F	**+**		**+**			**+**	
10	3 Years	F		**+**	**+**			**+**	
11	2 Years	F		**+**	**+**			**+**	
12	6 Years	F			**+**				**+**
13	4 Years	F			**+**				
14	5 Years	F		**+**	**+**				
15	3 Years	F			**+**				
16	4 Years	F			**+**				
17	5 Years	F		**+**	**+**				
18	4 Months	F		**+**	**+**	**+**	**+**		
19	4 Months	F		**+**	**+**	**+**	**+**		**+**
20	4 Months	M	**+**	**+**	**+**	**+**	**+**		**+**
21	4 Months	F	**+**		**+**				
22	3 Months	F		**+**	**+**				
23	8 Days	F	**+**		**+**				
24	1 Month	F	**+**		**+**				
25	1.5 Months	F	**+**		**+**				
26	3 Months	F	**+**		**+**				**+**
27	3 Months	F	**+**		**+**				**+**
28	2 Months	F	**+**		**+**				
29	1 Month	F	**+**		**+**				
30	2.5 Months	F	**+**		**+**				**+**
31	1 Month	F	**+**		**+**				
32	3.5 Months	F	**+**		**+**				

F, Female; M, Male; NL, Lymph node; **+**, Organ with lesions suggestive of tuberculosis.

**Fig 1:**
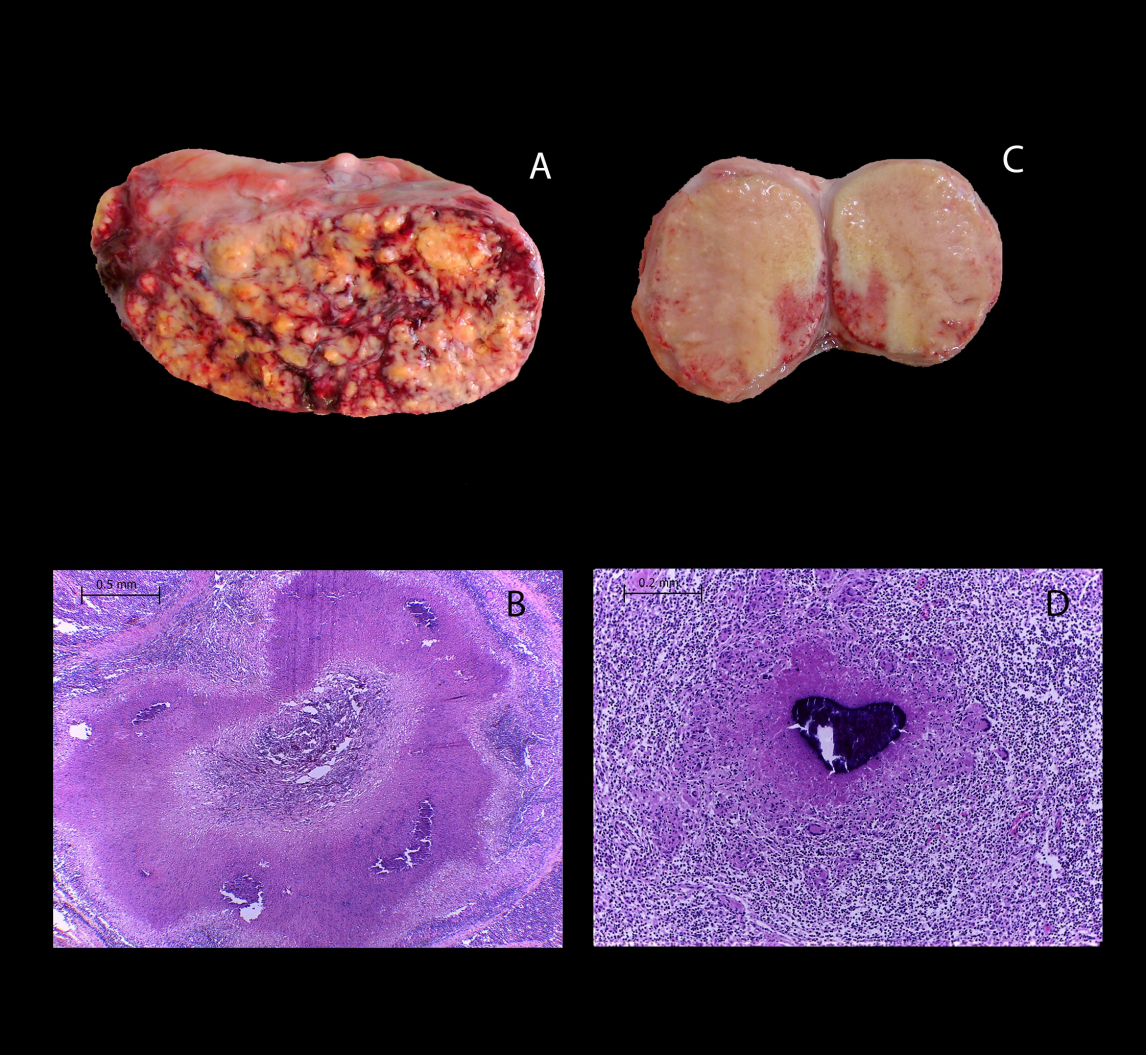
Extensive lesions suggestive of tuberculosis were observed in lymph nodes from calves naturally infected with *M*. *bovis and* compared to lesions found in adult cattle. **(A)** Mediastinal lymph node with severe granulomatous lymphadenitis and **(B)** H&E, 40x showing a granuloma encapsulated by connective tissue and abundant necrosis and mineralization from a 3-year-old Holstein-Friesian dairy cow. **(C)** Mediastinal lymph node with extensive areas of caseous necrosis and microhemorrhages and **(D)** H&E, 40x showing a granuloma without connective tissue and abundant necrosis and mineralization from a one-month-old Holstein-Friesian calf.

Different lesion degrees were identified by gross pathology in mediastinal lymph node approximately 2-fold large than the normal ones as well a slight increase in size of a lymph node was observed in some instances, without apparent pathological changes when sectioned lesions observed in the adult animal group, aggregates of epithelioid cells were however identified histologically, with intercalating lymphocytes and multinucleated giant cells. Small foci of multifocal calcification were observed in other cases, as well as necrotic areas with abundant caseous material corresponding microscopically to granulomas with extensive necrosis, mineralization and abundant connective tissue (**Fig 1A and 1B**). Interestingly, lymph nodes presenting lesions suggestive of tuberculosis were approximately 2 to 5-fold larger in calves than those found in adult animals. Cut surface of affected organs showed multiple poorly delimited lesions with extensive white-caseous necrotic areas throughout the exposed anatomical plane. Histologically, extensive areas of necrosis and calcification were observed **(Fig 1C and 1D)**. Intriguingly, 80% (12/15) of the calves showed extensive granulomatous lesions characterized by solid nodules, which had multiple white-caseous necrotic surface areas that were poorly demarcated and not surrounded by a connective tissue capsule. Also, coalescence between lesions could be observed **(S1 Fig).** In 23.5% (4/17) of animals with affected lungs, granulomatous lesions were identified both in pleura and parenchyma. Although lesion search followed pathologic protocols, presence of additional minute lesions in lung tissues cannot be ruled out.

### Granulomas in calves have abundant acid-fast bacilli (AFB)

**Fig 2:**
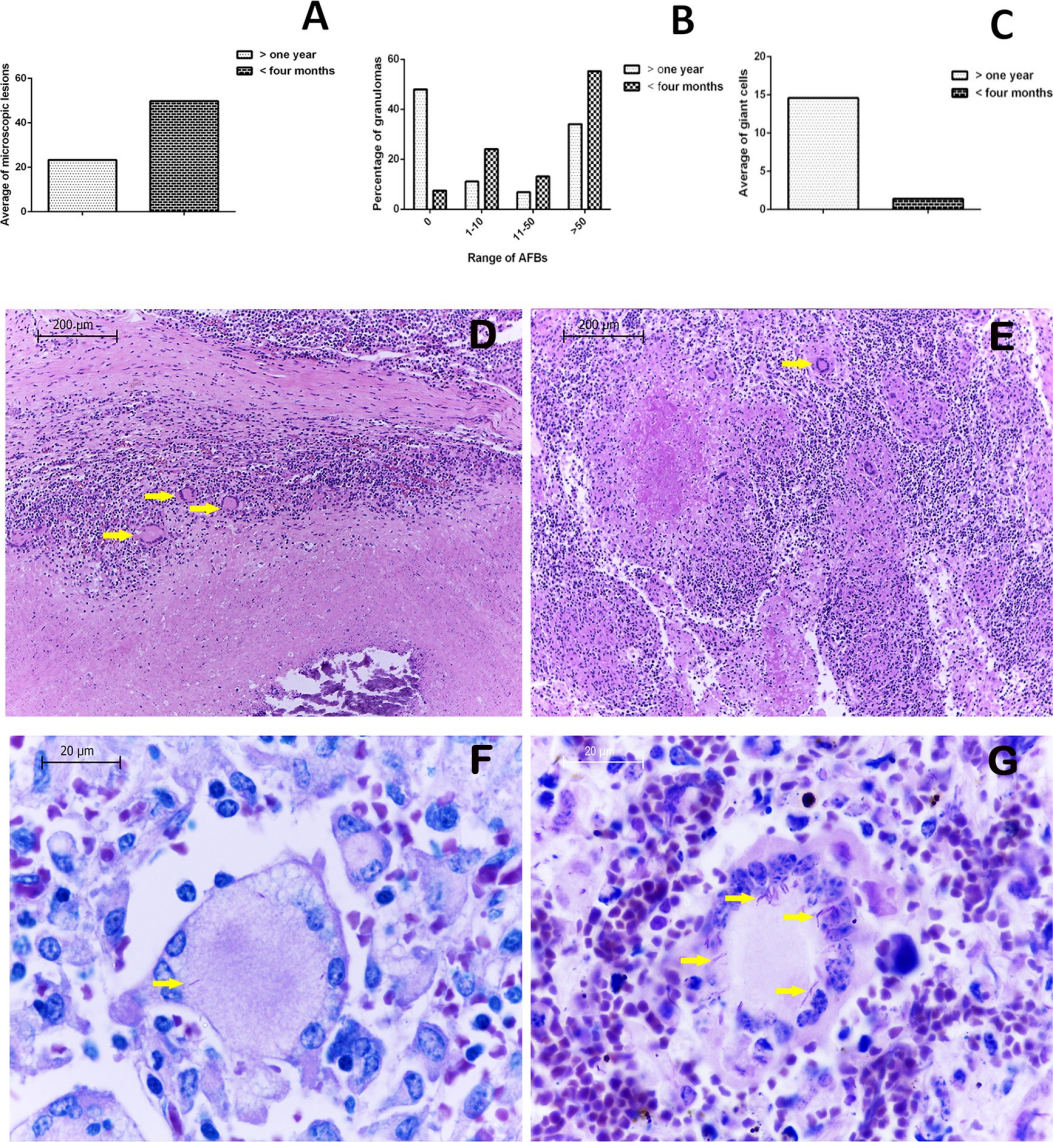
Calves had a greater number of granulomatous lesions and a higher bacilli scale when compared to adults. **(A)** Average microscopic lesions in cattle from both groups. **(B)** Granulomas with more than 50 bacilli are frequently seen in calves. **(C)** A lower average of giant cells is observed in calf lesions. **(D)** H&E, 40x, showing abundant giant cells (arrows) located between necrosis and fibrous capsule of a stage IV granuloma in adult cattle. **(E)** H&E, 400x, showing giant cells (arrows) close to an area with calcium deposits within a calf granuloma. Zielh Neelsen Stain (ZN) **(F and G 1000x) (F)** ZN stain showing a giant cell with intracytoplasmic bacilli in an adult bovine granuloma, and **(G)** ZN stain from a calf granulomatous lesion, showing a giant cell with abundant bacilli marked with arrows.

A total of 203 FFPE tissues were microscopically analyzed. On average, 6.3 FFPE samples with their corresponding H&E stained slides were evaluated per animal. From the 1143 identified lesions suggestive of tuberculosis, 54.06% were found in mediastinal lymph nodes, in agreement with a greater number of macroscopic lesions being found in the same location. A greater number of lesions was observed in calves (65.35%, 747/1143), with an average of 49.8 granulomas per case, while the remaining 34.64% of lesions (396/1143) were found in adult cattle, with an average of 23.2 identified granulomas per animal **(Fig 2A)**. In both groups a statistically significant association of a greater number of lesions with AFB was observed in comparison with the lesions without bacilli. Stage I had the highest percentage of lesions with bacilli in 41.47% (372/897) and the lowest was the stage III 9.7% (20/206) in adults and stage III-IV 9.8% (68/691) in calves (P = 0.0001). In addition, a greater number of AFB was observed within calf lesions, (55.28% of lesions with more than 50 bacilli, compared to 34.09% found in older cattle). Conversely, adult animals had a higher percentage of lesions without detectable AFB (47.9%), in contrast to only 7.4% found in calves **(Fig 2B).** Interestingly, identified granulomas from young animal tissues showed a lower number of multinucleated giant cells when compared to adult cattle (an average of 1.4 *vs* 14.5 cells per lesion respectively) **(Fig 2C–2E).** In addition, more bacilli were seen within giant cells from younger animal lesions **(Fig 2F and 2G).**

### Calcium deposits in granulomatous lesions caused by *M*. *bovis* are independent of connective tissue capsule presence in calves

**Fig 3:**
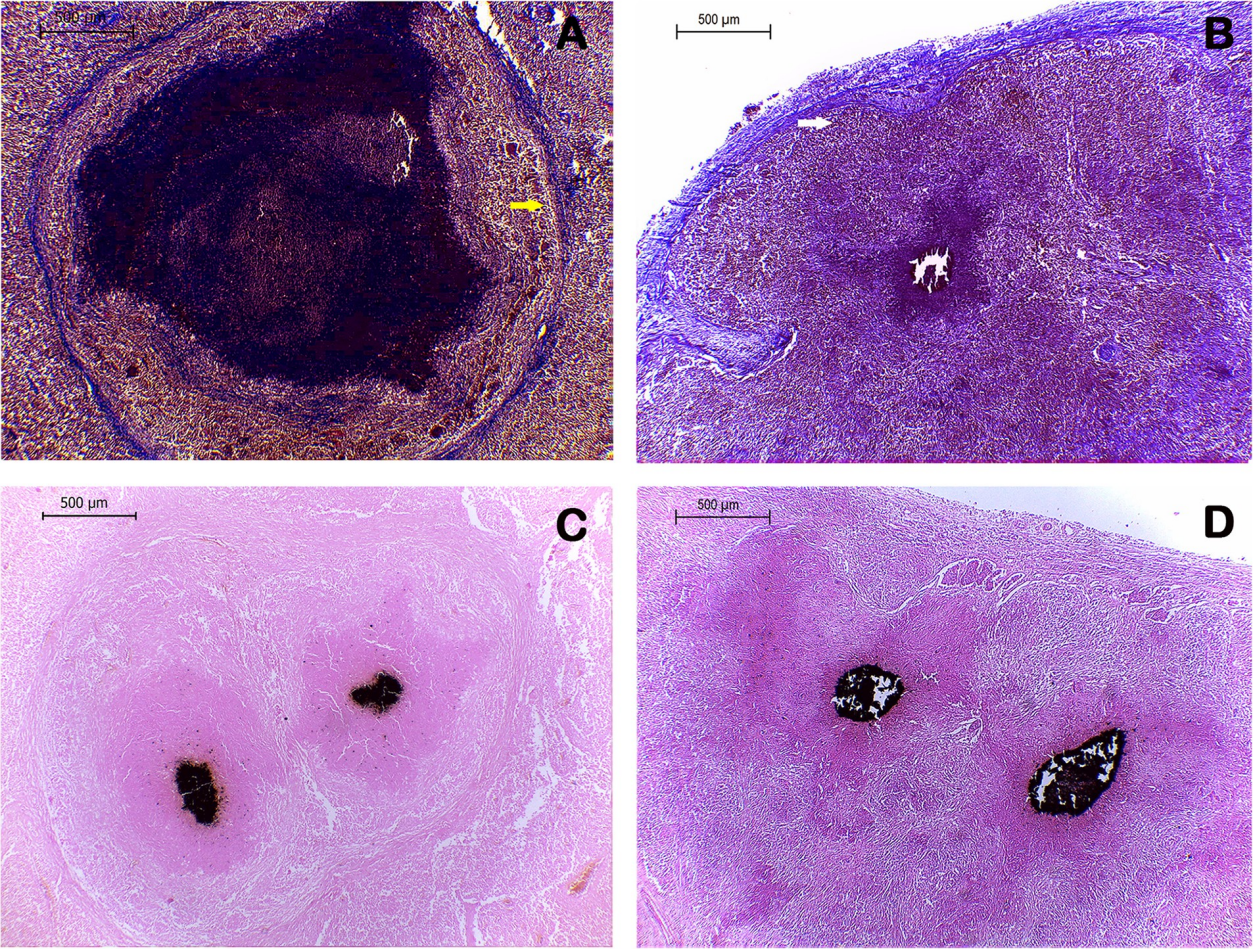
Calcium deposits in granulomatous lesions are independent of fibrous capsule presence in calves. **(A)** Masson's trichrome stained section showing a stage IV granuloma surrounded by abundant fibrous tissue (yellow arrow) in a bovine adult. **(B)** Masson's trichrome stained section showing a stage III-IV granuloma, without a fibrous capsule, but with surrounding disorganized collagen fibers in a calf. White arrow indicates connective tissue of the lymph node capsule as a reference. **(C and D)** Von Kossa staining, 200x, exhibiting stage IV (adult) and stage III-IV (calf) granulomatous lesions, with calcium deposits dyed in black.

In adult cattle, classic granulomatous lesions showing necrotic areas with calcium deposits and a thick capsule of connective tissue surrounding the lesion were observed. However, granulomas identified in calves lacked a fibrous capsule. Absence of capsule was confirmed by Masson's trichrome staining. Indeed, no fibrous capsule surrounding any lesion from calf tissues was observed, although some granulomas showed disorganized fibroblasts within necrotic areas **(Fig 3A).** Calcium deposits were observed within necrotic areas of granulomas in both animal groups as black colored precipitates by Von Kossa staining. Remarkably, abundant areas of black color were seen even in the absence of fibrosis in some lesions from calves **(Fig 3C and 3D).**

### Calves four months of age or younger showed atypical granulomas

**Fig 4:**
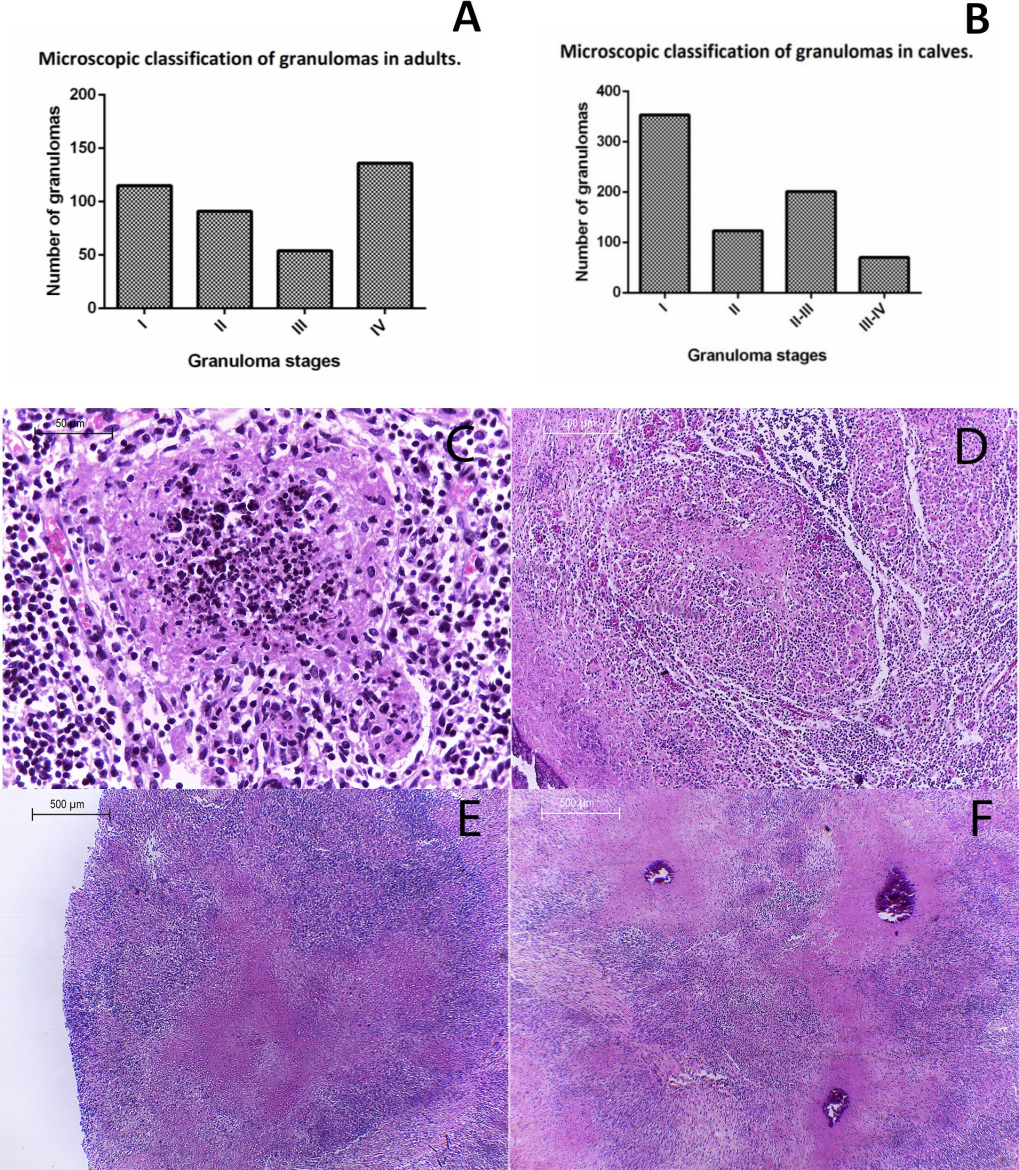
Histopathological classification of granulomas found in lymph nodes from calves four months of age or younger. **(A)** Graph of granuloma stages identified in adults, showing a higher frequency of stages I and IV. **(B)** Graph showing a higher frequency of stage I granulomas in calves. H&E (**C) Stage I:** 400X, small diffuse foci of infiltrated inflammatory cells, mainly macrophages, epithelioid macrophages and abundant cellular detritus at the center of the lesion. **(D) Stage II:** 200X, Larger structure with epithelioid macrophages, multinucleated giant cells, cell debris and necrosis. **(E) Stage II-III:** 40X, large necrotic areas without borders, absence of connective tissue capsule or lymphocyte accumulation in the periphery. **(F) Stage III-IV**: 40X, lesions showing extensive necrotic areas, mineralization and cell debris without defined borders, surrounded by macrophages, absence of connective tissue capsule or lymphocytes in the periphery; lesions tend to coalesce and fuse.

All identified granulomatous lesions from the adult cattle group (396 in total), were classified: Stage I (initial) is characterized by aggregates of epithelioid macrophages, presence of giant Langerhans cells with interspersed lymphocytes, no necrosis and occasional neutrophil infiltrate; Stage II (Solid), mainly composed of epithelioid macrophages, there is a greater number of multinucleated giant cells than in the previous stage, lymphocyte presence and minimal caseous necrosis at the center of the lesion. Stage III (minimal necrosis), granulomas are fully encapsulated, with well-developed caseous necrosis and minimal mineralization. Stage IV (necrosis and mineralization), granulomas are surrounded by a thick capsule of connective tissue, there is extensive necrosis with areas of mineralization and adjoining epithelioid macrophages, presence of lymphocytes and multinucleated giant cells [8]. Most granulomatous lesions from adult cattle were classified as stage IV (34.3%, 136/396), presenting abundant connective tissue and calcification as shown by Masson's Trichrome and Von Kossa stains respectively. Stage I Granulomas (29.0%, 115/396) were mainly found as satellite lesions to more advanced stages (III and IV). Stage III granulomas were the least frequently observedv13.6% (54/396) (**Fig 4A**). Our results show a positive correlation between granuloma stage and giant cell number (r = 0.74, p = 0.0001). Indeed, stage IV granulomas had the greater number of giant cells with an average of 32.9 cells/granuloma, followed by stage III lesions with an average of 12.4 cells, stage II with 3.8 cells and finally the lowest number of giant cells was found in stage I granulomas (0.6 giant cells/ lesion).

A total of 747 granulomatous lesions suggestive of tuberculosis were identified by histopathology in different tissues from calves. Lungs were the most affected organs (49.6% of the lesions, 371/747), whereas only 4.9% of lesions were found in mesenteric lymph nodes (37/747).

**Fig 5:**
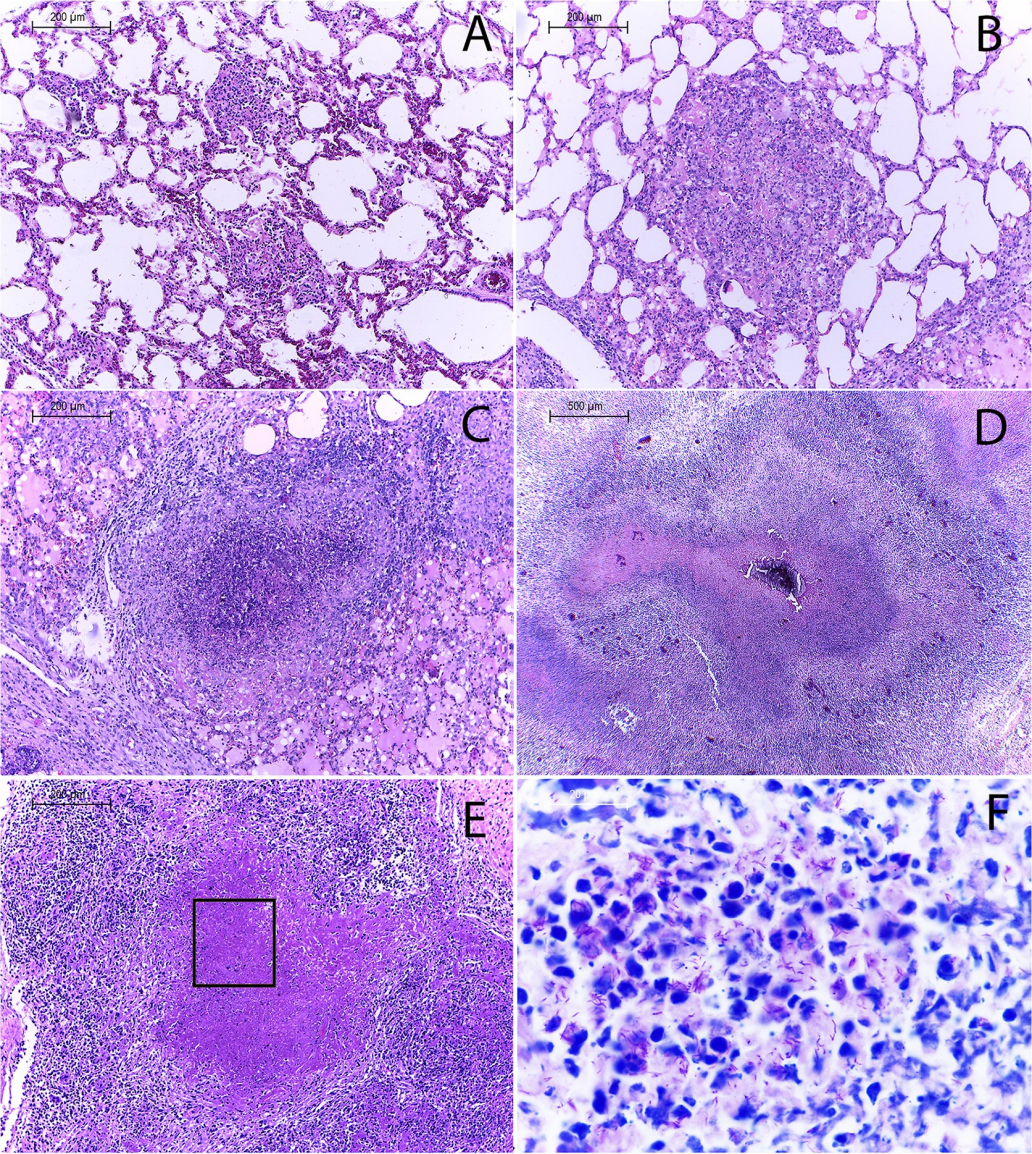
Histopathological classification of granulomas found in lung tissue from calves. H&E (**A.-C** 200x, **D-E** 40x **F.-**1000x). **(A)** Stage I. **(B)** Stage II. **(C)** Stage II-III. **(D)** Stage III-IV. **(E)** Close up of stage II-III. **(F)** Zielh Neelsen 1000x with abundant extracellular acid-fast bacilli in stage II-III.

Since characteristics of microscopic lesions found in animals four months of age or younger did not fully comply with the developmental stage classification proposed by Wangoo *et al*. (2005) [8], revisions were made to categorize lymph node and lung lesions in four modified stages designated as I, II, II-III and III-IV. Following this classification, stage I was the most frequently observed developmental degree of lesions from young animal tissues (47.2%, 353/747); whereas stage III-IV was the least frequently detected (9.3%, 70/747) **(Fig 4B).** Our suggested classification for granulomatous lesions from calves is briefly described below:

**Stage I**: Buildup of epithelioid macrophages, absence of capsule, central necrosis with cellular debris; neutrophils can be present **(Figs 4C and 5A)**. **Stage II**: Numerous epithelioid macrophages with lymphocyte infiltrate; occasional giant cells and extensive necrosis and cellular debris **(Figs 4D and 5B)**. **Stage II-III:** Extensive necrosis and cellular debris with poorly delimited borders, absence of a fibrotic capsule or adjoining peripheral lymphocytes, and a few macrophages or giant cells in the periphery **(Figs 4E, 5C, 5E and 5F)**. **Stage III-IV:** Extensive necrosis with calcification and cell debris without defined borders; numerous peripheral macrophages, absence of fibrous capsule and few lymphocytes; lesions tend to coalesce **(Figs 4F and 5D).**

### Bacteriological isolation and molecular genotyping of mycobacterial infection by PCR from tissues presenting lesions suggestive of tuberculosis in naturally infected animals

**Fig 6:**
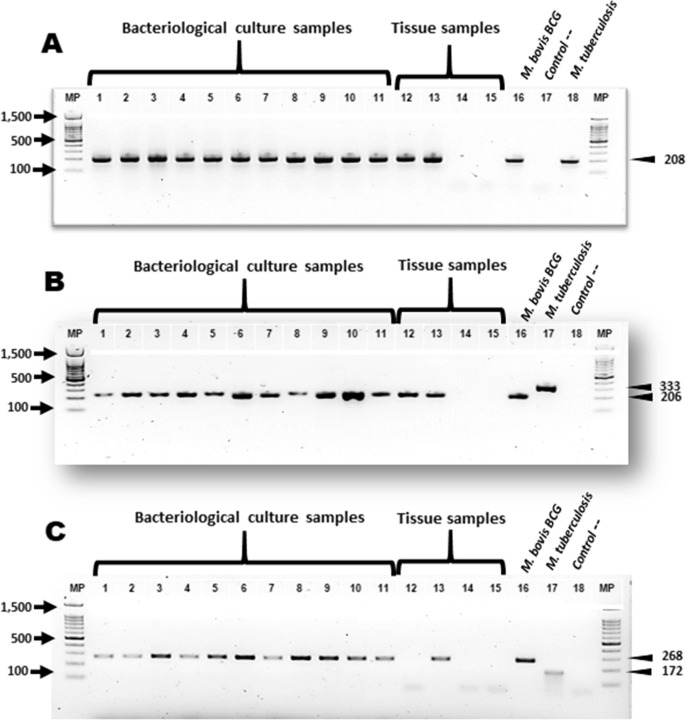
Molecular identification of *M*. *bovis* in naturally infected cattle. **(A)** Nested PCR amplification products of the 208 bp mpb70 gene. **(B)** Endpoint PCR of the 206 bp fragment from the *M*. *bovis* RD9 gene and the 333 bp fragment from the *M*. *tuberculosis* gene. **(C)** Endpoint PCR of the 268 bp fragment from the *M*. *bovis* RD4 gene and the 172 bp fragment from the *M*. *tuberculosis* gene **MP** molecular weight marker is 100 bp; lanes 1–11: bacteriological culture samples; lanes 12–15: negative tissue samples; and lanes 16–18: positive controls (*M*. *bovis* BCG and *M*. *tuberculosis* H37rv) and negative control.

Suggestive growth of *Mycobacterium spp*. was observed in 55% (11/20) of the processed samples. Subsequently, we performed DNA extraction and assessed by PCR for molecular genotyping of *M*. *bovis* of: eleven isolates, from nine tissues of culture-negative cases and twelve FFPE tissues. All these cases presented macroscopic and microscopic lesions compatible with tuberculosis, as well as the presence of AFB. The mpb70 / m22, RD9 and RD4 genes were used for PCR amplification. Of these 84.3% (27/32) amplified a product of genes Mpb70 / m22 and RD9. Product amplification of gene RD4 was possible in 46.8% (15/32) samples (**Table 2**) and (**Fig 6A and 6B**).

The deletion of RD9 is observed not only in *M*. *africanum* and *M*. *bovis* but also in other mycobacteria like *M*. *caprae*, *M*. *microti* and *M*. *canettii*. However, with the exception of *M*. *bovis* those species have never been reported in Mexico.

**Table 2:** Bacteriological isolation and molecular genotyping of *M*. *bovis*.

Case number	Age	Sex	Histology compatible with tuberculosis	Presence of AFB	Bacteriological isolation	PCR genes		
						MPB70/M22	RD9	RD4
1	4 Years	F	**+**	**+**	**−**	**+**	**+**	**−**
2	4 Years	F	**+**	**+**	**−**	**+**	**+**	**+**
3	2 Years	F	**+**	**+**	**+**	**+**	**+**	**+**
4	3 Years	F	**+**	**+**	**−**	**−**	**−**	**−**
5	3 Years	F	**+**	**+**	**+**	**+**	**+**	**+**
6	5 Years	F	**+**	**+**	**−**	**−**	**−**	**−**
7	5 Years	F	**+**	**+**	**−**	**+**	**+**	**+**
8	3 Years	F	**+**	**+**	**+**	**+**	**+**	**+**
9	6 Years	F	**+**	**+**	**+**	**+**	**+**	**+**
10	3 Years	F	**+**	**+**	**+**	**+**	**+**	**+**
11	2 Years	F	**+**	**+**	**+**	**+**	**+**	**+**
12	6 Years	F	**+**	**+**	**+**	**+**	**+**	**+**
13	4 Years	F	**+**	**+**	**−**	**−**	**−**	**−**
14	5 Years	F	**+**	**+**	**−**	**+**	**+**	**−**
15	3 Years	F	**+**	**+**	**−**	**+**	**+**	**−**
16	4 Years	F	**+**	**+**	**−**	**+**	**+**	**−**
17	5 Years	F	**+**	**+**	**+**	**+**	**+**	**+**
18	4 Months	F	**+**	**+**	**+**	**+**	**+**	**+**
19	4 Months	F	**+**	**+**	**+**	**+**	**+**	**+**
20	4 Months	M	**+**	**+**	**+**	**+**	**+**	**+**
21	4 Months	F	**+**	**+**	**N/A**	**+**	**+**	**−**
22	3 Months	F	**+**	**+**	**N/A**	**+**	**+**	**+**
23	8 Days	F	**+**	**+**	**N/A**	**+**	**+**	**+**
24	1 Month	F	**+**	**+**	**N/A**	**−**	**−**	**−**
25	1.5 Months	F	**+**	**+**	**N/A**	**−**	**−**	**−**
26	3 Months	F	**+**	**+**	**N/A**	**+**	**+**	**−**
27	3 Months	F	**+**	**+**	**N/A**	**+**	**+**	**−**
28	2 Months	F	**+**	**+**	**N/A**	**+**	**+**	**−**
29	1 Month	F	**+**	**+**	**N/A**	**+**	**+**	**−**
30	2.5 Months	F	**+**	**+**	**N/A**	**+**	**+**	**−**
31	1 Month	F	**+**	**+**	**N/A**	**+**	**+**	**−**
32	3.5 Months	F	**+**	**+**	**N/A**	**+**	**+**	**−**

F, Female; M, Male; N/A, not applicable; AFB, Acid-fast bacilli; **+**, positive result; **−**, negative result.

## Discussion and conclusions

There are several studies in cattle experimentally infected by *M*. *bovis* that have focused on describing anatomic and pathological characteristics, including distribution of lesions and histological distinction of granulomas. However, fewer studies have detailed descriptions of granulomatous lesions in naturally infected cattle. In this study, lesions suggestive of tuberculosis from 32 Holstein-Friesian cattle naturally infected by *M*. *bovis* were macroscopically and microscopically evaluated. Considering characteristics of lesions, results were divided in two groups according to animal age, where the adult cattle group included animals over one year old (53.2%, 17/32 animals), while the young animal group included calves four months of age or younger (46.8%, 15/32 animals). Interestingly, gross pathology assessment of lesions from lungs and lymph nodes from calves showed extensive consolidation with coalescent necrotic areas. Further histopathological evaluation evidenced extensive necrosis, calcification and absence of a connective tissue capsule, as well as abundant mainly extracellular acid-alcohol resistant bacilli. These findings can relate to previous reports where granulomatous lesions in lungs, liver and lymph nodes from calves under 45 days old and naturally infected by *M*. *bovis* where severely disseminated with extensive necrotic areas. Abundant necrosis with AFB presence in lung lesions was also microscopically identified [19–20].

Bovine tuberculosis is considered a disease that mainly affects the airways. In naturally infected cattle, lungs and associated lymph nodes are the most frequently concerned organs at post-mortem examination [20]. This study shows that mediastinal lymph nodes were involved in 100% (32/32) of the animals that presented lesions suggestive of tuberculosis, and lungs were affected in 50% of cattle (16/32). These results agree with previous reports and underline the importance of aerial passages as pathways for mycobacterial entry. A thorough pathological analysis of lungs was performed in this study, however due to the large size of these organs, some lesions could have been missed. In the four months of age or younger group 80% of animals (12/15) showed lung lesions that were easily detected macroscopically. More than 50% of the lung parenchyma was affected in some instances, likely indicating the airways as route of infection. Frequency of lung involvement in calves was unexpected since it is commonly thought that young animal lesions are mainly found in mesenteric lymph nodes consequent to intake of contaminated milk [21]. In this study, 33.3% (5/15) of calves presented lesions in mesenteric lymph nodes, and 80% (4/5) of which also had affected lungs. Therefore, possibility of a simultaneous infection through both routes cannot be ruled out. Nonetheless, *M*. *bovis* can also be transmitted through the congenital route, which has been previously reported in 15 to 25-day old calves [19–21]. Detection of granulomatous lesions in a young 8-day-old calf included in this study, could indicate congenital transmission as possible rout of infection. Therefore, bovine tuberculosis should be included when performing a differential diagnosis in young animal necropsies [21].

Different developmental stages of granuloma formation could be found within the same organ, during histological assessment of tissue sections in this study. Presence of different microenvironments within the same tissue has been previously suggested in studies with cattle experimentally infected with *M*. *bovis* [22–23]. A total of 396 microscopically identified granulomas from the adult animal group, were categorized [8]. Most were stage IV lesions, surrounded by stage I satellite granulomas. In this study, 136 stage IV granulomas were identified in adults, of which 63.9% (87/136) did not present AFB and only 17.64 (24/136) had more than 50 bacilli per granuloma. Stage IV lesions have been previously associated with a large number of bacilli in experimental infections with *M*. *bovis*. Conversely, in naturally infected adult animals, presence of a connective tissue capsule surrounding lesions could indicate a better control of disease development by the host [22,24–27].

Predominance of stage IV granulomas in naturally infected animals indicate a chronic process involving an anti-inflammatory immune response, that can also be related to the fibrosis that is observed surrounding lesions. Macroscopical and microscopical characteristics of lesions found in calves differed from those observed in adult tissues, precluding granuloma classification for the former age group according to stages set by Wangoo *et al*., (2005) [8]. Revised the established categories, suggesting four modified stages (I, II, II-III and III-IV) to classify calf lesions. According to this new classification, most of the identified granulomas in calf tissues were stage I lesions (47.2%, 353/747); whereas stage III-IV granulomas were the least frequent (9.3%, 70/747). Stage III-IV lesions showed calcification and absence of a fibrous capsule. The average number of giant cells per lesion was 1.4 compared to 14.5 cells/granuloma in stage IV lesions observed in adult cattle.

Interestingly, atypical pattern of granulomatous lesion formation has also been reported in in C3HeB/FeJ mice infected with *M*. *bovis*. In these rodents, lesions were characterized by extensive necrotic areas, presence of neutrophils, disorganized fibrous capsule formation, and abundant bacilli that caused death by 5 weeks post-infection [28]. Lesions that develop after natural infection of young cattle with *M*. *bovis*, also present traditional markers of acute infection, such as neutrophil presence and an exacerbated pro-inflammatory response. However, repeatability of these results needs to be validated in further studies under different conditions. Nonetheless, we submit an adapted classification for stages of granuloma formation in young animals naturally infected with *M*. *bovis*.

In conclusion, this study identified a large number of granulomatous lesions in mediastinal lymph nodes and lungs of cattle naturally infected with *M*. *bovis*, suggesting the airways as the main route of entry for mycobacteria during natural infection. Stage IV granulomas are the most frequently found lesion in naturally infected animals over one year old, in accordance with the chronic nature of the disease. Finally, calves four months of age or younger presented lesions with atypical macroscopic and microscopic characteristics, which were more abundant and had a greater number of associated bacilli when compared to adult cattle. These findings contribute to better understand the physiopathology of *Mycobacterium bovis* infection in cattle.

## Supporting information

S1 Fig Severe granulomatous pneumonia in calves. Lung surface sections from calves aged four months or younger with granulomatous pneumonia, showing extensive white areas lacking delimited edges, which may coalesce.
(TIF)
