# Supplementary figures and images for: Atypical granuloma formation in Mycobacterium bovis-infected calves
Source: PLoS One. 2019 Jul 15;14(7):e0218547. doi: 10.1371/journal.pone.0218547 (PMC6629060; doi:10.1371/journal.pone.0218547)

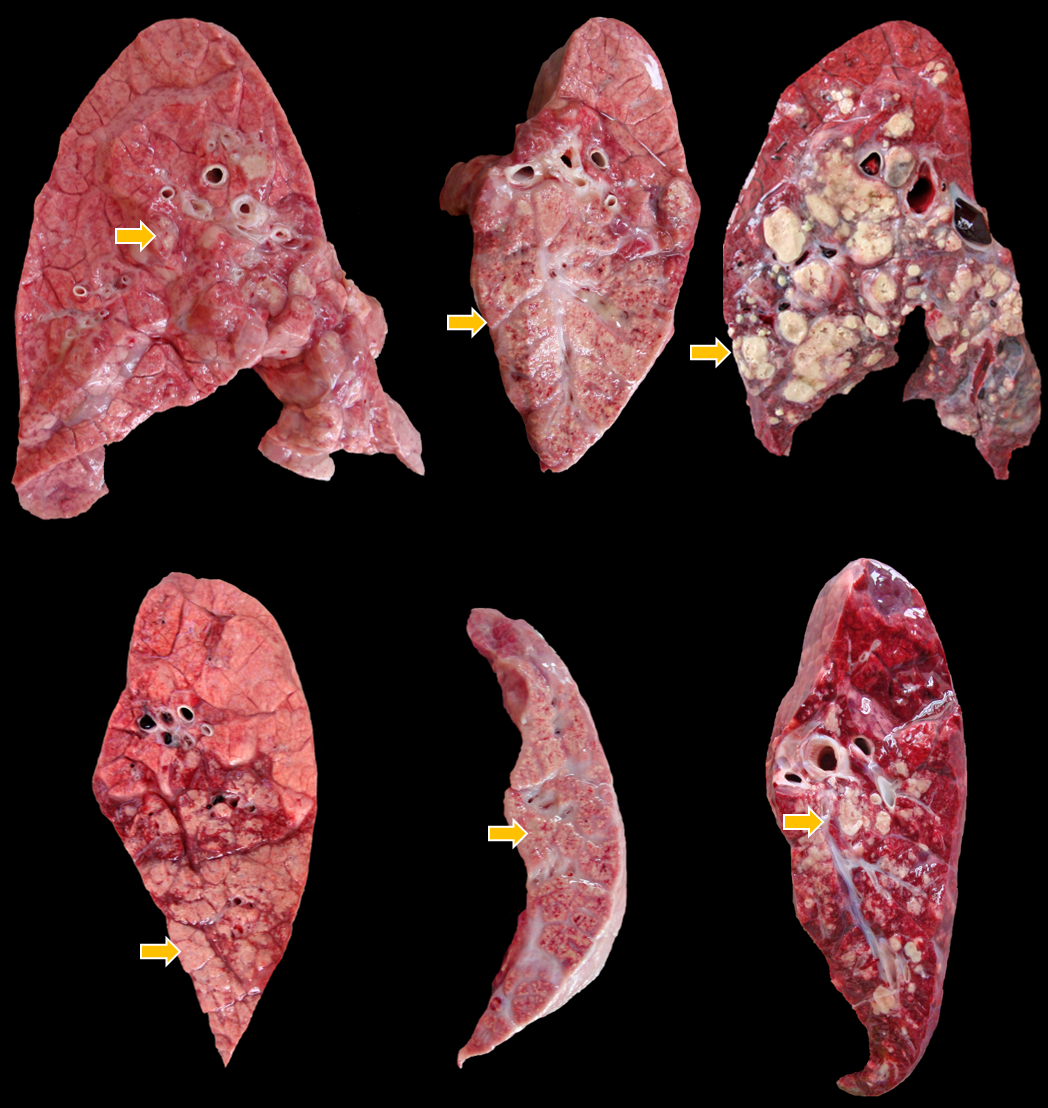

Supplement: S1 Fig — Lung surface sections from calves aged four months or younger with granulomatous pneumonia, showing extensive white areas lacking delimited edges, which may coalesce. (TIF) [file pone.0218547.s001.tif]
